# RNA Editing Genes Associated with Extreme Old Age in Humans and with Lifespan in *C. elegans*

**DOI:** 10.1371/journal.pone.0008210

**Published:** 2009-12-14

**Authors:** Paola Sebastiani, Monty Montano, Annibale Puca, Nadia Solovieff, Toshio Kojima, Meng C. Wang, Efthymia Melista, Micah Meltzer, Sylvia E. J. Fischer, Stacy Andersen, Stephen H. Hartley, Amanda Sedgewick, Yasumichi Arai, Aviv Bergman, Nir Barzilai, Dellara F. Terry, Alberto Riva, Chiara Viviani Anselmi, Alberto Malovini, Aya Kitamoto, Motoji Sawabe, Tomio Arai, Yasuyuki Gondo, Martin H. Steinberg, Nobuyoshi Hirose, Gil Atzmon, Gary Ruvkun, Clinton T. Baldwin, Thomas T. Perls

**Affiliations:** 1 Department of Biostatistics, Boston University School of Public Health, Boston, Massachusetts, United States of America; 2 Department of Medicine Sections of Infectious Diseases, Boston University School of Medicine, Boston, Massachusetts, United States of America; 3 Department of Genetics, IRCCS Multimedica, Milan, Italy; 4 Computational Systems Biology Research Group, Advanced Science Institute, RIKEN, Yokohama, Kanagawa, Japan; 5 Department of Genetics, Massachusetts General Hospital and Harvard Medical School, Boston, Massachusetts, United States of America; 6 Center for Human Genetics, Boston University School of Medicine, Boston, Massachusetts, United States of America; 7 Geriatrics Section, Department of Medicine, Boston University School of Medicine, Boston, Massachusetts, United States of America; 8 Department of Bioengineering, Boston University School of Engineering, Boston, Massachusetts, United States of America; 9 Division of Geriatric Medicine, Department of Internal Medicine, Keio University School of Medicine, Tokyo, Japan; 10 Department of Systems & Computational Biology, Albert Einstein College of Medicine, Bronx, New York, United States of America; 11 Institute of Aging Research and Diabetes Research Center, Departments of Medicine and Genetics, Albert Einstein College of Medicine, Bronx, New York, United States of America; 12 Department of Molecular Genetics and Microbiology, University of Florida at Gainesville, Florida, United States of America; 13 Department of Pathology, Tokyo Metropolitan Geriatric Hospital, Tokyo, Japan; 14 Clinical Thanatology and Geriatric Behavioral Science, Graduate School of Human Sciences, Osaka University, Suita, Osaka, Japan; Brunel University, United Kingdom

## Abstract

**Background:**

The strong familiality of living to extreme ages suggests that human longevity is genetically regulated. The majority of genes found thus far to be associated with longevity primarily function in lipoprotein metabolism and insulin/IGF-1 signaling. There are likely many more genetic modifiers of human longevity that remain to be discovered.

**Methodology/Principal Findings:**

Here, we first show that 18 single nucleotide polymorphisms (SNPs) in the RNA editing genes *ADARB1* and *ADARB2* are associated with extreme old age in a U.S. based study of centenarians, the New England Centenarian Study. We describe replications of these findings in three independently conducted centenarian studies with different genetic backgrounds (Italian, Ashkenazi Jewish and Japanese) that collectively support an association of *ADARB1* and *ADARB2* with longevity. Some SNPs in *ADARB2* replicate consistently in the four populations and suggest a strong effect that is independent of the different genetic backgrounds and environments. To evaluate the functional association of these genes with lifespan, we demonstrate that inactivation of their orthologues *adr-1* and *adr-2* in *C. elegans* reduces median survival by 50%. We further demonstrate that inactivation of the argonaute gene, *rde-1*, a critical regulator of RNA interference, completely restores lifespan to normal levels in the context of *adr-1* and *adr-2* loss of function.

**Conclusions/Significance:**

Our results suggest that RNA editors may be an important regulator of aging in humans and that, when evaluated in *C. elegans*, this pathway may interact with the RNA interference machinery to regulate lifespan.

## Introduction

Exceptional longevity (EL) in humans, defined as living to extreme old ages such as 100 years and older, is strongly familial [1]–[8] and the factors that facilitate such exceptional survival have broad public health significance including a marked delay in age-related disability [9]–[11] and certain age-related diseases [12]–[14]. Genetically, exceptional longevity is presumed to be a complex trait [15]–[19]. Several candidate gene association studies have been successful in discovering longevity-associated genes in humans. However, these variants have been mainly related to lipoprotein metabolism [20]–[22], FOXO proteins [23], [24], and insulin/IGF-1 signaling [25] It is likely that many more genetic modifiers of human aging have yet to be discovered [25].

In this study, we investigate two genes in the A(adenosine) to I (inosine) RNA editing pathway, a post-transcriptional process by which adenosine residues are converted to inosine resulting in a change in gene expression or protein function. Targets of RNA editing include a large number of genes as well as micro RNA. Thus, it is not surprising that such a non-specific cellular process would be involved in a general maintenance of cellular health and lifespan. However, such an implication has not been previously demonstrated.

Here, we first report significant association of these genes with EL in four centenarian studies that include the New England Centenarian Study (NECS), with more than 1,500 US individuals of primarily North European ancestry, aged between 90 and 119 years; the Southern Italian Centenarian Study (SICS) –a study of nonagenarians and centenarians from a closed population of Cilento, South Italy; the Ashkenazi Jewish Centenarian Study (AJCS), a study of approximately 300 nonagenarians and centenarians from a founder population of North Eastern European background, all resident in the US; and the Japanese Centenarian Study (JCS), a study of Japanese centenarians that has focused on “semi-supercentenarians” subjects living past 105 years [26]. The characteristics of the four populations allow us to assess the robustness of the associations to varying genetic background and environment.

We further evaluate the functional significance of the RNA editing candidate genes in *C. elegans* lifespan studies and show that silencing orthologs of these genes reduces median survival by 50%. We also show that life span is fully restored by additional knockdown of an RNA interference gene, supporting the functional role of these genes in determining lifespan and implicating a novel axis for future aging studies.

## Results and Discussion

### Selection of Candidate Genes

We selected the two genes to study for multiple reasons. First, in a preliminary genome wide screening using pooled DNA samples from approximately 130 male centenarians and 130 younger male controls from the NECS [27], we identified several single nucleotide polymorphisms (SNPs) in the RNA-editing genes *ADARB1* (21q22.3), and *ADARB2* (10p15.3) that were associated with extreme old age. *ADARB1* exhibited the strongest evidence for genetic association with 5 SNPs that met genome-wide significance, with the posterior odds of allelic association >1,500 [27]. The probability of these 5 SNPs simultaneously associated under the null hypothesis of no association was 10^−13^ based upon a hyper-geometric distribution. Second, this gene lies in chromosome 21q21 and trisomy 21 (Down syndrome) resembles accelerated aging, with premature age-related changes including in the skin and hair, increased frequency of premature cataracts, hearing loss, menopause and Alzheimer's disease [28] suggesting that genes in chromosome 21 could affect lifespan. Third, among the top genes identified from the preliminary genetic screen, RNA editing represents a general cellular process that might be expected to improve cellular health; and RNA editing activity has been associated with innate immune response [29], [30] and age-related syndromes that include dementia and amyotrophic lateral sclerosis (ALS) [31].

### Subjects Selected for the Association Study

From the NECS, genotype data were obtained from 281 males, aged 96–114 years and 596 females, aged 100–119 years. We selected cutoff ages of 96 years for males and 100 years for females of the NECS to focus on the extreme top 1% survival based on the U.S. Social Security Administration cohort life table (http://www.ssa.gov/OACT/NOTES/as116/as116LOT.html). NECS referent cohort subjects consisted of 270 spouses of centenarian offspring and children of parents who died at the mean age of 73 years (average life expectancy for the parents' birth cohort). Additional referent subjects were selected from the Illumina iControlDB database using genome-wide genetic matching as detailed in the methods (n = 1635). Note that approximately 100 male centenarians included in the pooling-based genome screening overlap with this second set. Given that the overlap is relatively small (<10%) and that the subsequent analysis uses a different analytic approach (genotype data from individual subjects), we do not think the overlap is a significant concern.

From the SICS, we used genotype data from 271 males, ages 90–108 years and 188 females, aged 90–109 years (total = 459). Data from 200 male and 132 female SICS referent cohort subjects aged 18–48 years were used in this analysis. From the AJCS, genotype data were obtained from 299 oldest subjects (108 males aged 95 and older and 191 females aged 99 and older) and 269 younger referent cohort subjects (spouses of the offspring of centenarians, aged 85 and younger, without evidence of parental longevity). Four hundred and seventy oldest old subjects (82 males aged 100–110 years and 388 females, aged 100–116 years) and 538 referent cohort subjects (randomly selected Japanese subjects, aged 19–89 years) constituted the Japanese association study. Table 1 reports further summaries of subjects' characteristics. Ages of the extreme old were validated with birth certificates (in the case of the JCS, the Basic Resident Registration Card). All subjects were enrolled by studies with Institutional Review Board approval and oversight.

**Table 1:** Study Subjects characteristics.

	Males	Females	All	Males	Females	All
	**NECS oldest old**			**NECS controls**		
**Sample Size**	**281**	**596**	**877**	**149**	**121**	**270**
**Median Age**	**102**	**103**	**103**	**75**	**74**	**75**
**Age Range**	**96–114**	**100–119**	**96–119**	**58–85**	**53–85**	**53–85**
	**SICS oldest old**			**SICS controls**		
**Sample Size**	**271**	**188**	**459**	**200**	**132**	**332**
**Median Age**	**94**	**98**	**96**	**34**	**32**	**33**
**Age Range**	**90–109**	**90–109**	**90–109**	**18–48**	**18–48**	**18–48**
	NA			**Illumina controls**		
**Sample Size**				**418**	**1217**	**1635**
**Median Age**				**47**	**46**	**47**
**Age Range**				**30–75**	**30–75**	**30–75**
	**AJCS oldest old**			**AJCS controls**		
**Sample Size**	**108**	**191**	**299**	**118**	**151**	**269**
**Median Age**	**99**	**101**	**100**	**77**	**73**	**73**
**Age Range**	**95–108**	**99–112**	**95–112**	**54–85**	**46–85**	**46–85**
	**JCS oldest old**			**JCS controls**		
**Sample Size**	**82**	**388**	**470**	**178**	**360**	**538**
**Median Age**	**104**	**106**	**106**	**21**	**72**	**69**
**Age Range**	**100–111**	**100–116**	**100–116**	**19–89**	**19–89**	**19–89**

Reported are summaries of the last contact ages.

### Association of ADARB1 with Exceptional Longevity

**Figure 1:**
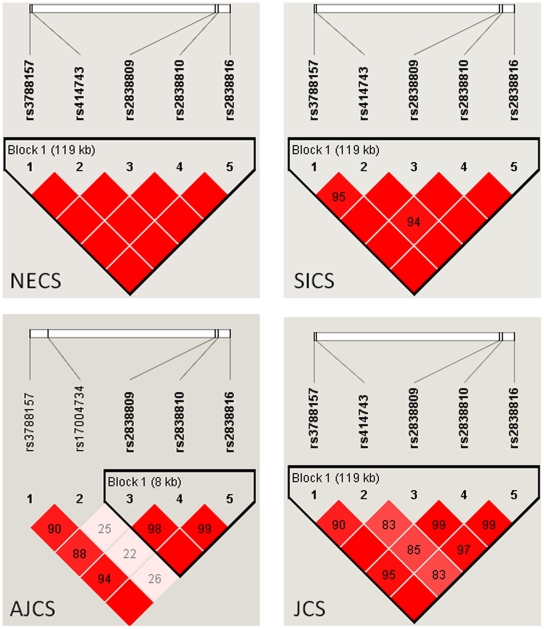
Pattern of LD among the SNP in *ADARB1* (chromosome 21) that are associated with exceptional longevity. The four plots display the pattern of LD captured by the SNPs associated with exceptional longevity in *ADARB1* (chromosome 21) using data from the NECS, SICS, AJCS and JCS. The intensity of red represents the strength of LD measured by D′.

**Figure 2:**
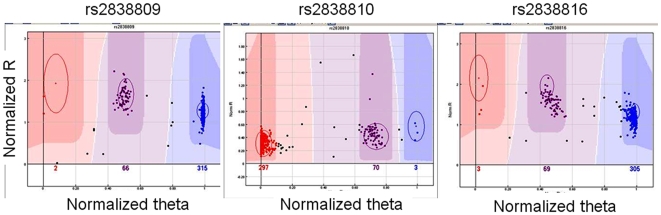
Result of genotype cluster algorithm from BeadStudio. The three plots show the normalized intensities in polar coordinate and the cluster definition from BeadStudio for NECS subjects. The clear separation suggests that the genotype calls are robust.

We examined the associations of 31 SNPs in *ADARB1* in the NECS and SICS samples using recessive and dominant models with Bayesian logistic regression [32]. The details of the statistical analysis are in the Methods and the significant results are summarized in Table 2 that provides the physical positions and allele frequencies derived from the HapMap for these SNPs, and Table 3 (rows 14–15). Five SNPs in *ADARB1* are strongly associated with extreme old age in the NECS, and the association of SNP rs414743 remains significant even after imposing stringent corrections for multiple comparisons (Bayesian significance <0.05/145 = 0.00035 where 145 is the overall number of SNPs included in this analysis). The five SNPs tag one region of strong linkage disequilibrium (LD) of the gene (Figure 1). None of the SNPs reached statistical significance in the SICS although the three SNPs rs2838809, rs2838810 and rs2838816 exhibited consistent associations in terms of odds ratios and allele frequencies and, when the NECS and SICS data were combined, the three SNPs remained statistically significant. These three SNPs have extreme minor allele frequencies in the NECS centenarians (MAF<0.01), while the allele frequencies in the controls are very close to referent allele frequencies from the HapMap (Table 3). Figure 2 displays the scatter plot of genotype intensities generated from BeadStudio that rules out genotyping errors thus suggesting that these are real associations and not artifacts.

**Table 2:** Summary of selected SNPs.

Row	SNP	Chr	position	Risk versus referent alleles	CEPH	JPT
**1**	**rs10903420**	**10**	**1333726**	AA v AG/GG	0.327	0.058
**2**	**rs1007147**	**10**	**1341088**	AA v AG/GG	0.312	0.091
**3**	**rs2805562**	**10**	**1357514**	AA v AG/GG	0.15	0.058
**4**	**rs884949**	**10**	**1361610**	AA v AC/CC	0.124	0
**5**	**rs2805533**	**10**	**1374633**	AA/AG v GG	0.77	0.92
**6**	**rs2387653**	**10**	**1397826**	AA v AG/GG	0.097	0
**7**	**rs2805535**	**10**	**1450432**	AA v AG/GG	0.699	0.151
**8**	**rs2805543**	**10**	**1454892**	AA v AG/GG	0.46	0.105
**9**	**rs3898610**	**10**	**1474759**	AA v AG/GG	0.841	0.686
**10**	**rs1533484**	**10**	**1481339**	AA/AG v GG	0.442	0.791
**11**	**rs2676192**	**10**	**1495474**	AA v AG/GG	0.301	0.419
**12**	**rs2387952**	**10**	**1657365**	AA v AG/GG	0.69	0.616
**13**	**rs17294019**	**10**	**1659347**	AA/AG v GG	0.196	0.012
**14**	**rs3788157**	**21**	**45335136**	AA v AG/GG	0.63	0.65
**15**	**rs414743**	**21**	**45336503**	AA/AG v GG	0.47	0.31
**16**	**rs2838809**	**21**	**45445866**	AA v AG/GG	0.009	0
**17**	**rs2838810**	**21**	**45447751**	AA/AG v GG	1	1
**18**	**rs2838816**	**21**	**45454470**	AA v AG/GG	0.009	0

List of 18 SNPs —13 in the gene *ADARB2* (10p15.3) and 5 in the gene *ADARB1* (21q22.3) — that are associated with exceptional longevity with either dominant or recessive models. The table reports a sequential number for easy identification in the other tables and figures (column 1), the SNP identifier from dbSNP (column 2), chromosome and physical position from the human genome NCBI Build 36.3 (columns 3 and 4), the risk versus referent alleles that were associated with exceptional longevity using dominant and recessive models (column 5), the frequencies of the risk allele in the HapMap CEPH and JPT. Note that several of alleles in the Japanese group have allele frequencies that change substantially from the CEPH, and the SNPs in rows 4 and 6 become monomorphic.

**Table 3:** SNPs in *ADARB2* (10p15.3) and *ADARB1* (21q22.3) that are associated with exceptional longevity in NECS and SICS subjects.

	Row	SNP	Risk versus referent alleles	CEPH	NECS (877 oldest old, 1808 controls)			SICS (459 oldest old, 429 controls)			NECS+SICS		
					OR	Bayes sig	p(A)	OR	Bayes sig	p(A)	OR	Bayes sig	p(A)
**ADARB2**	**1**	**rs10903420**	AA v AG/GG	0.327	**1.28(1.07;1.53)**	**0.0048**	0.28/0.23	1.14(0.84;1.54)	0.1212	0.28/0.25	**1.25(1.07;1.45)**	**0.0020**	0.28/0.24
	**2**	**rs1007147**	AA v AG/GG	0.312	**1.35(1.13;1.64)**	**0.0015**	0.27/0.22	1.25(0.92;1.66)	0.2040	0.29/0.25	**1.34(1.15;1.59)**	**0.0003**	0.27/0.22
	**3**	**rs2805562**	AA v AG/GG	0.15	**1.22(0.96;1.54)**	**0.0500**	0.15/0.12	**1.38(0.97;1.97)**	**0.0384**	0.20/0.15	**1.32(1.09;1.59)**	**0.0022**	0.16/0.13
	**4**	**rs884949**	AA v AC/CC	0.124	1.19(0.92;1.53)	0.0911	0.12/0.10	1.31(0.87;1.96)	0.0978	0.14/0.11	**1.24(1.01;1.54)**	**0.0211**	0.12/0.10
	**5**	**rs2805533**	AA/AG v GG	0.77	0.91(0.75;1.12)	0.1904	0.78/0.81	0.83(0.60;1.14)	0.1217	0.76/0.79	**0.86(0.72;1.02)**	**0.0381**	0.79/0.81
	**6**	**rs2387653**	AA v AG/GG	0.097	1.17(0.91;1.50)	0.1053	0.14/0.12	1.12(0.78;1.62)	0.2699	0.170.16	**1.21(0.99;1.49)**	**0.0343**	0.15/0.13
	**7**	**rs2805535**	AA v AG/GG	0.699	**1.36(1.02;1.83)**	**0.0249**	0.75/0.69	**1.31(0.97;1.79)**	**0.0395**	0.72/0.66	**1.37(1.11;1.68)**	**0.0017**	0.74/0.68
	**8**	**rs2805543**	AA v AG/GG	0.46	**1.23(1.04;1.45)**	**0.0055**	0.54/0.49	**1.36(1.03;1.76)**	**0.0156**	0.51/0.43	**1.24(1.08;1.42)**	**0.0008**	0.53/0.48
	**9**	**rs3898610**	AA v AG/GG	0.841	**1.42(1.11;1.77)**	**0.0015**	0.88/0.83	1.19(0.80;1.74)	0.1973	0.87/0.85	**1.36(1.12;1.67)**	**0.0015**	0.87/0.84
	**10**	**rs1533484**	AA/AG v GG	0.442	**0.81(0.70;0.97)**	**0.0109**	0.35/0.40	**0.79(0.63;1.01)**	**0.0428**	0.43/0.49	**0.86(0.75;0.98)**	**0.0147**	0.38/0.42
	**11**	**rs2676192**	AA v AG/GG	0.301	**0.81(0.67;0.98)**	**0.0096**	0.26/0.30	0.95(0.74;1.31)	0.3869	0.26/0.27	**0.83(0.71;0.96)**	**0.0086**	0.26/0.30
	**12**	**rs2387952**	AA v AG/GG	0.69	**1.37(1.15;1.66)**	**0.0004**	0.75/0.69	1.05(0.80;1.37)	0.3484	0.66/0.65	**1.20(1.04;1.40)**	**0.0073**	0.72/0.68
	**13**	**rs17294019**	AA/AG v GG	0.196	**0.69(0.55;0.86)**	**0.0005**	0.14/0.19	0.80(0.59;1.10)	0.0826	0.22/0.27	**0.78(0.65;0.93)**	**0.0030**	0.17/0.21
**ADARB1**	**14**	**rs3788157**	AA v AG/GG	0.63	**1.23(1.05;1.45)**	**0.0075**	0.65/0.62	0.90(0.68;1.19)	0.2318	0.65/0.67	**1.16(1.00;1.34)**	**0.0204**	0.65/0.62
	**15**	**rs414743**	AA/AG v GG	0.47	**0.72(0.61;0.85)**	**<10−5**	0.40/0.48	1.08(0.83;1.45)	0.2689	0.49/0.46	**0.83(0.72;0.96)**	**0.0053**	0.43/0.48
	**16**	**rs2838809**	AA v AG/GG	0.009	**0.27(0.05;0.93)**	**0.0096**	0.003/0.009	0.58(0.15;1.97)	0.1973	0.011/0.016	**0.46(0.19;1.08)**	**0.0297**	0.005/0.01
	**17**	**rs2838810**	AA/AG v GG	1	**3.71(1.12;19.29)**	**0.0136**	0.997/0.990	2.29(0.63;10.33)	0.1088	0.991/0.984	**2.73(1.11;8.25)**	**0.0136**	0.995/0.99
	**18**	**rs2838816**	AA v AG/GG	0.009	**0.28(0.05;0.93)**	**0.0116**	0.003/0.009	0.58(0.15;1.98)	0.1934	0.011/0.016	**0.48(0.18;1.04)**	**0.0309**	0.005/0.01

The first 13 SNPs are in the gene *ADARB2* (10p15.3) and the last five SNPs in the gene *ADARB1* (21q22.3). Columns 1–4 provide details of the SNPs as in table 2. Columns 5–7 report the results of the association in the NECS and referent subjects based on Bayesian logistic regression of dominant and recessive models. Specifically, column 5 reports the Bayesian estimate of the odds ratio and 95% credible interval within brackets, columns 6 reports the Bayes significance that is defined as 1-p(OR>1) when the posterior estimate of the OR is >1 and 1-p(OR<1) when the posterior estimate of the OR is <1. This number is the posterior probability of the null hypothesis OR≤1 when we estimate OR>1 (or OR≥1 when we estimate OR<1) so small values provide strong evidence against the null hypothesis and it is similar to the Bayes p-value proposed by Althman. Column 7 reports the posterior probability of the risk allele in cases and controls. Highlighted in bold are the significant associations (Bayes significance <0.05). Columns 8–10 report the replications in the SICS and highlighted in bold are the 4 SNPs that are significant in this analysis. Columns 11–12 report the results of the analysis when the data from the two studies are aggregated. Although only 15 SNPs reach statistical significance in the NECS and only 4 SNPs in the SICS, all 18 have consistent effects and when data of the two studies are aggregated, all SNPs remain significant and the significance of 11 of them becomes stronger.

**Figure 3:**
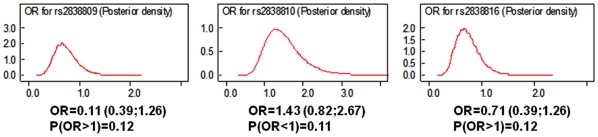
Posterior densities of ORs. Posterior densities of the ORs for the 3 SNPs in ADARB1 with rare alleles and moderate effects in the data aggregated from NECS, SICS and JCS. Significant associations would results in posterior densities not overlapping 1 and definite evidence of either an OR<1 or an OR>1, while all of the three densities have heavy tails and do not provide definite evidence against the null hypothesis of no association.

To further test the generalizability of these results to other independent groups, we evaluated these associations in the AJCS and the JCS, using a combination of proxy SNPs typed with the Affymetrix platforms and SNPs in Table 3 typed with more traditional techniques (See methods). Table 4 summarizes the results of the replication study of 4 of the 5 SNPs and one additional proxy SNP is in Table 5. None of these SNPs in *ADARB1* replicates the results in the NECS and SICS samples although the significant association of the SNP rs17004734 that is within 2Kb from rs414743 is consistent with the presence of longevity associated variants in the region. Because the SNPs used in the NECS and SICS are chosen to best capture the genetic variations of Caucasians from the HapMap, they may not be the correct choice for this founder population and indeed Figure 1 shows a different pattern of LD in *ADARB1* in the AJCS subjects. All *ADARB1* SNPs in Table 3 and two additional proxy SNPs were genotyped in the JCS subjects and Tables 6 and 7 summarize the results. The last three SNPs in Table 6 show effects that are consistent with the NECS and SICS subjects but do not reach statistical significance, even when the data from the three studies are aggregated. Figure 3 shows the posterior densities of the ORs for the three rare SNPs that are suggestive of association but would need much larger sample sizes to reach statistical significance. The association of two proxy SNPs for rs2838816 in Table 7 is again consistent with the presence of longevity associated variants in this gene that may not be captured by our SNP selection in the Japanese population.

**Table 4:** Replication in the AJCS subject set.

	Row	SNP	Risk versus referent alleles	CEPH	NECS+SICS			AJCS (299 oldest old, 269 controls)			NECS+SICS+AJCS		
					OR	Bayes sig	p(A)	OR	Bayes sig	p(A)	OR	Bayes sig	p(A)
**ADARB2**	**1**	**rs10903420**	AA v AG/GG	0.327	**1.25(1.07;1.45)**	**0.0020**	0.28/0.24	1.37(0.87;2.18)	0.0861	0.27/0.21	**1.26(1.09;1.45)**	**0.0010**	0.28/0.24
	**2**	**rs1007147**	AA v AG/GG	0.312	**1.34(1.15;1.59)**	**0.0003**	0.27/0.22						
	**3**	**rs2805562**	AA v AG/GG	0.15	**1.32(1.09;1.59)**	**0.0022**	0.16/0.13	**1.88(1.10;3.22)**	**0.0095**	0.21/0.17	**1.39(1.16;1.65)**	**<10−5**	0.17/0.13
	**4**	**rs884949**	AA v AC/CC	0.124	**1.24(1.01;1.54)**	**0.0211**	0.12/0.10						
	**5**	**rs2805533**	AA/AG v GG	0.77	**0.86(0.72;1.02)**	**0.0381**	0.79/0.81	0.73(0.45;1.18)	0.0955	0.76/0.81	**0.84(0.72;0.98)**	**0.0129**	0.78/0.81
	**6**	**rs2387653**	AA v AG/GG	0.097	**1.21(0.99;1.49)**	**0.0343**	0.15/0.13	1.40(0.83;2.38)	0.1030	0.20/0.15	**1.27(1.05;1.53)**	**0.0082**	0.17/0.13
	**7**	**rs2805535**	AA v AG/GG	0.699	**1.37(1.11;1.68)**	**0.0017**	0.74/0.68	1.27(0.82;1.95)	0.1476	0.81/0.77	**1.28(1.06;1.54)**	**0.0050**	0.73/0.70
	**8**	**rs2805543**	AA v AG/GG	0.46	**1.24(1.08;1.42)**	**0.0008**	0.53/0.48	1.26(0.85;1.87)	0.1217	0.51/0.45	**1.22(1.08;1.39)**	**0.0001**	0.52/0.47
	**9**	**rs3898610**	AA v AG/GG	0.841	**1.36(1.12;1.67)**	**0.0015**	0.87/0.84	0.63(0.35;1.16)	0.0685	0.87/0.91	**1.29(1.07;1.55)**	**0.0035**	0.87/0.84
	**10**	**rs1533484**	AA/AG v GG	0.442	**0.86(0.75;0.98)**	**0.0147**	0.38/0.42						
	**11**	**rs2676192**	AA v AG/GG	0.301	**0.83(0.71;0.96)**	**0.0086**	0.26/0.30	0.77(0.52;1.15)	0.0986	0.27/0.32	**0.83(0.72;0.95)**	**0.0037**	0.26/0.30
	**12**	**rs2387952**	AA v AG/GG	0.69	**1.20(1.04;1.40)**	**0.0073**	0.72/0.68	0.95(0.61;1.44)	0.3977	0.67/0.69	**1.17(1.01;1.34)**	**0.0146**	0.71/0.68
	**13**	**rs17294019**	AA/AG v GG	0.196	**0.78(0.65;0.93)**	**0.0030**	0.17/0.21	**1.42(0.95;2.13)**	**0.0451**	0.31/0.24			
**ADARB1**	**14**	**rs3788157**	AA v AG/GG	0.63	**1.16(1.00;1.34)**	**0.0204**	0.65/0.62	**0.73(0.50;1.05)**	**0.0463**	0.58/0.65			
	**15**	**rs414743**	AA/AG v GG	0.47	**0.83(0.72;0.96)**	**0.0053**	0.43/0.48						
	**16**	**rs2838809**	AA v AG/GG	0.009	**0.46(0.19;1.08)**	**0.0297**	0.005/0.01	1.19(0.44;3.29)	0.3662	0.040/0.034			
	**17**	**rs2838810**	AA/AG v GG	1	**2.73(1.11;8.25)**	**0.0136**	0.995/0.99	0.99(0.39;2.53)	0.4951	0.956/0.956			
	**18**	**rs2838816**	AA v AG/GG	0.009	**0.48(0.18;1.04)**	**0.0309**	0.005/0.01	1.06(0.38;3.06)	0.4573	0.035/0.034			

List of the SNPs in *ADARB2* and *ADARB1* that reach statistical significance in the NECS and SICS and were attempted to be replicated in the AJCS set. The first 7 columns report the details of the SNPs as described in the legend of Table 3. Columns 8–11 report the results for 14 of the 18 SNPs in Table 3 that were genotyped in 255 oldest old and 227 younger controls of the AJCS. Only 3 SNPs reach statistical significance in this set (rows 3, 13 and 14) but two of them have opposite effects compared to the NECS and SICS (rows 13 and 14). However, SNPs in rows 1, 5–9, 11 and 12 have effects that are consistent with the NECS and SICS and, when the data of the 3 studies are aggregated, they become significant (columns 12–14). The SNPs in rows 2, 4, 10 and 11 were not typed because proxy SNPs from an ongoing genome wide association study conducted with the Affymetrix 6.0 array suggest strong associations in the same region.

**Table 5:** Replication in the AJCS subject set using proxy SNPs.

Row	SNP	Risk versus referent alleles	CEPH	NECS+SICS			Proxy SNPs				AJCS (255 oldest old, 227 controls)			Risk versus referent allele
				OR	Bayes sig	p(A)	SNP	Position	Distance	D′/r^2^	OR	Bayes sig	p(A)	
**2**	**rs1007147**	AA v AG/GG	0.312	**1.34(1.15;1.59)**	**0.0003**	0.27/0.22	**rs2804097**	**1352129**	**11041**	**0.63/0.3**	**0.56(0.33;0.91)**	**0.01**	0.76/0.85	AA/AT v TT
**4**	**rs884949**	AA v AC/CC	0.124	**1.24(1.01;1.54)**	**0.0211**	0.12/0.10	**rs10903426**	**1361386**	**−224**	**1.00/0.58**	**1.62(1.00;2.73)**	**0.0269**	0.23/0.16	CC v CT/TT
**15**	**rs414743**	AA/AG v GG	0.47	**0.83(0.72;0.96)**	**0.0053**	0.43/0.48	**rs17004734**	**45345886**	**2383**	**1.00/0.07**	**0.29(0.06;1.00)**	**0.0259**	0.95/0.98	AA/AG v GG

Additional proxy SNPs that were genotyped in the AJCS set and tag the same region as shown by their proximity in terms of physical distance <10kb and linkage disequilibrium measured by D′ and r^2^ (See also Figure 1).

**Table 6:** Replication in the JCS subject set.

	Row	SNP	Risk versus referent alleles	CEPH	NECS+SICS			JPT	JCS (470 oldest old, 538 controls)		
					OR	Bayes sig	p(A)		OR	Bayes sig	p(A)
**ADARB2**	**1**	**rs10903420**	AA v AG/GG	0.327	**1.25(1.07;1.45)**	**0.0020**	0.28/0.24	0.058	**0.51(0.23;1.07)**	**0.0397**	0.03/0.045
	**2**	**rs1007147**	AA v AG/GG	0.312	**1.34(1.15;1.59)**	**0.0003**	0.27/0.22	0.091	1.10(0.68;1.81)	0.3435	0.08/0.075
	**3**	**rs2805562**	AA v AG/GG	0.15	**1.32(1.09;1.59)**	**0.0022**	0.16/0.13	0.058	0.67(0.26;1.56)	0.1813	0.02/0.03
	**4**	**rs884949**	AA v AC/CC	0.124	**1.24(1.01;1.54)**	**0.0211**	0.12/0.10	0			
	**5**	**rs2805533**	AA/AG v GG	0.77	**0.86(0.72;1.02)**	**0.0381**	0.79/0.81	0.92	**0.59(0.37;0.92)**	**0.0110**	0.88/0.93
	**6**	**rs2387653**	AA v AG/GG	0.097	**1.21(0.99;1.49)**	**0.0343**	0.15/0.13	0			
	**7**	**rs2805535**	AA v AG/GG	0.699	**1.37(1.11;1.68)**	**0.0017**	0.74/0.68	0.151	1.02(0.69;1.49)	0.4690	0.14/0.14
	**8**	**rs2805543**	AA v AG/GG	0.46	**1.24(1.08;1.42)**	**0.0008**	0.53/0.48	0.105	0.86(0.57;1.30)	0.2381	0.10/0.12
	**9**	**rs3898610**	AA v AG/GG	0.841	**1.36(1.12;1.67)**	**0.0015**	0.87/0.84	0.686	1.09(0.83;1.43)	0.2619	0.66/0.64
	**10**	**rs1533484**	AA/AG v GG	0.442	**0.86(0.75;0.98)**	**0.0147**	0.38/0.42	0.791	**0.79(0.58;1.07)**	**0.0641**	0.76/0.80
	**11**	**rs2676192**	AA v AG/GG	0.301	**0.83(0.71;0.96)**	**0.0086**	0.26/0.30	0.419	0.92(0.71;1.19)	0.2542	0.40/0.42
	**12**	**rs2387952**	AA v AG/GG	0.69	**1.20(1.04;1.40)**	**0.0073**	0.72/0.68	0.616	0.98(0.76;1.28)	0.4576	0.59/0.60
	**13**	**rs17294019**	AA/AG v GG	0.196	**0.78(0.65;0.93)**	**0.0030**	0.17/0.21	0.012	0.54(0.12;2.03)	0.1846	0.01/0.02
**ADARB1**	**14**	**rs3788157**	AA v AG/GG	0.63	**1.16(1.00;1.34)**	**0.0204**	0.65/0.62	0.65	1.03(0.79;1.36)	0.4080	0.67/0.66
	**15**	**rs414743**	AA/AG v GG	0.47	**0.83(0.72;0.96)**	**0.0053**	0.43/0.48	0.31	1.14(0.86;1.52)	0.183	0.29/0.26
	**16**	**rs2838809**	AA v AG/GG	0.009	**0.46(0.19;1.08)**	**0.0297**	0.005/0.01	0	0.81(0.36;1.81)	0.2959	0.028/0.034
	**17**	**rs2838810**	AA/AG v GG	1	**2.73(1.11;8.25)**	**0.0136**	0.995/0.99	1	1.14(0.50;2.60)	0.3791	0.974/0.970
	**18**	**rs2838816**	AA v AG/GG	0.009	**0.48(0.18;1.04)**	**0.0309**	0.005/0.01	0	0.81(0.36;1.79)	0.3032	0.029/0.034

Lists of the SNPs in *ADARB2* and *ADARB1* that reach statistical significance in the NECS and SICS and were attempted to be replicated in the JCS set. The first 7 columns report the details of the SNPs as described in the legend of Table 3. Columns 8–11 report the results for 14 of the 18 SNPs in Table 3 that were genotyped in 470 oldest old and 538 younger controls of the JCS. Only 3 SNPs reach statistical significance in this set (rows 1, 5 and 10) but one of them have opposite effects compared to the NECS and SICS (row 1). We did not attempt to merge the results from different populations because of the substantial differences in allele frequencies.

**Table 7:** Replication in the JCS subject set using proxy SNPs.

Row	SNP	Chr	position	NECS+SICS			SNP	position	Distance	D′/r2	JPCS (432 oldest old, 346 controls)			Risk versus referent alleles
				OR	Bayes sig	p(A)					OR	Bayes sig	p(A)	
2	rs1007147	10	1341088	1.34(1.15;1.59)	0.0003	0.27/0.22	rs2804099	1346320	5232	0.63/0.3	**0.61(0.34;1.07)**	**0.0422**	0.91/0.94	CC/CG v GG
4	rs884949	10	1361610	1.24(1.01;1.54)	0.0211	0.12/0.10	rs10903426	1361386	−224	1.00/0.58	**1.70(0.97;3.05)**	**0.0332**	0.09/0.06	CC v CT/TT
6	rs2387653	10	1397826	1.22(0.98;1.48)	0.0343	0.15/0.13	rs17221652	1406472	8646	0.91/0.22	**1.84(1.01;3.58)**	**0.0234**	0.08/0.05	CC v CG/GG
7	rs2805535	10	1450432	1.37(1.11;1.68)	0.0017	0.74/0.68	rs4543904	1453158	2726	0.88/0.20	**0.44(0.23;0.80)**	**0.0031**	0.91/0.96	CC/CG v GG
18	rs2838816	21	45454470	0.48(0.18;1.04)	0.0309	0.005/0.01	rs6518219	45479145	24675	0.08/0.03	**1.36(1.03;1.82)**	**0.0202**	0.50/0.43	AA/AT v TT
							rs2838824	45479813	25343	0.08/0.03	**0.54(0.30;0.86)**	**0.0059**	0.89/0.93	CC/CT v TT

Additional SNPs that were genotyped in the JCS set and corroborate associations in the NECS and SICS subjects sets.

### Association of ADARB2 with Exceptional Longevity

**Figure 4:**
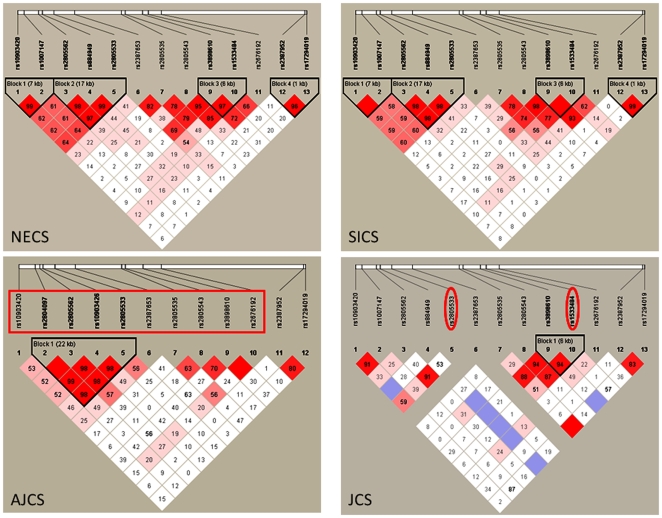
Pattern of LD among the SNP in *ADARB2* (chromosome 10) that are associated with exceptional longevity. The four plots display the pattern of LD captured by the SNPs associated with exceptional longevity in *ADARB2* (chromosome 10) in the NECS, SICS, AJCS and JCS data. The intensity of red cells represents the strength of LD measured by D′. The LD pattern in the NECS, SICS and AJCS subjects are very similar but differ substantially from the pattern of LD in the JCS subjects in which two SNPs become almost monomorphic (rs884949 and rs2387653). Highlighted in red are the SNPs that replicate the results in the AJCS and JCS subjects.

We examined the associations of 114 SNPs in *ADARB2* in the NECS and SICS samples using the same recessive and dominant models. Ten SNPs were strongly associated with extreme old age in the NECS, and one remains significant even after correcting for multiple comparisons (SNPs rs2387952, Bayesian significance 0.0004∼0.05/145). Four of these significant SNPs (rs2805562; rs2805533; rs2805543; and rs1533484) were also replicated in the SICS (Bayes significance <0.05) (Table 3). The remaining six SNPs did not reach statistical significance in the SICS but did exhibit consistent associations in terms of odds ratios and allele frequencies and combining data from the NECS and SICS made these ten associations even stronger plus an additional three other SNPs became statistically significant. These SNPs tag a region of approximately 160Kb in *ADARB2* that includes two blocks of LD (Figure 4).

Tables 4 and 5 summarize the results of the replication of 10 of these SNPs in AJCS subjects. Two of the SNPs reach statistical significance in this set (rows 3 and 13) but one has opposite effects compared to the NECS and SICS (rows 13). However, SNPs in rows 1, 5–9, 11 and 12 have effects that are consistent with the NECS and SICS and, when the data of the 3 studies are aggregated, they become significant (columns 12–14). The SNPs in rows 2, 4, and 11 were not typed but proxy SNPs typed with the Affymetrix 6.0 array confirm strong associations of variants in the same region with EL. These SNPs are summarized in Table 5 and are a good proxy for the SNPs originally typed in the NECS and SICS as shown by their proximity in terms of physical distance <10kb and linkage disequilibrium measured by D′ and r^2^.

Only SNP rs2805533 reached statistical significance in the JCS set, with an effect that was consistent with the observed effect in the NECS, SICS and AJCS samples (Table 6). The SNP rs1533484 was borderline significant (Bayesian significance ∼0.06) and demonstrates consistent effects with the NECS and SICS results, but the allele frequencies are substantially different. The SNP rs10903420 was also significant but with an opposite effect compared to the NECS, SICS and AJCS subject sets. Note however the substantial differences in both allele frequencies and pattern of LD that may explain the different patterns of associations in this ethnically very distinct sample. Genotype data of additional SNPs in Table 7 provide further evidence for the existence of variants in the region between physical positions 1340K and 1500K of chromosome 10 that are associated with exceptional longevity.

### Age Trends

**Figure 5:**
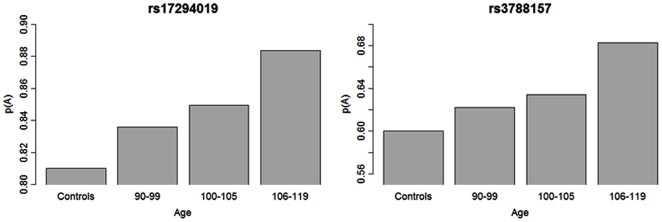
Age related trend of allele frequencies. The two barplots show the age related trend of allele frequencies of SNPs rs17294019 (*ADARB2*, SNP # 98 in Table 1) and rs3788157 (ADARB1, SNP # 135 in Table 1) in the NECS (n = 1,023). The frequencies of the common allele for both SNPs were stratified in the age groups 90–99; 100–105, 106 and higher. Trends of allele frequencies for increasing age groups are consistent with a strong correlation between genotype and phenotype that results in substantial enrichment of protective alleles in older subjects.

For some SNPs, a clear monotonic pattern associated with increasing age was observed (Figure 5). This monotonic pattern is consistent with a genetic effect that results from alleles positively associated with EL becoming more frequent in older individuals, while alleles that are negatively associated with EL become less frequent. This pattern has also been observed for ApoE alleles [33] and is consistent with the phenomenon of demographic selection [34]. These increases in allele frequencies with age also illustrate the increasing gain of power conferred by studying centenarians and even more so, subjects age 105+ years, in genetic studies of exceptional survival.

### Functional Studies

**Figure 6:**
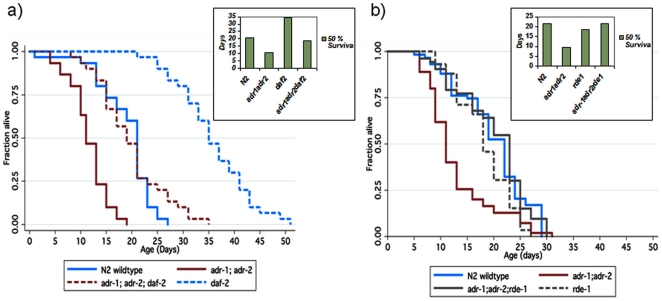
*ADAR* mediated decline in lifespan, *daf-2* influence, and *rde-1* rescue. a) Lifespan using mutant strains for *adr-1;adr-2* in the context of dsRNA mediated gene inactivation of *daf-2*. Synchronized worms at the larval stage 4 (L4) were sterilized with FudR and allowed to feed on bacterial lawns that contained dsRNA for *daf-2*. Note: *adr-1*; *adr-2* double mutant (red solid), *adr-1*; *adr-2* double mutant with dsRNA for *daf-2* (red hatched), N2 wild type (blue solid), N2 with dsRNA for *daf-2* (blue hatched). Note decline in lifespan due to *adr-1*; *adr-2* compared with N2 wildtype. Also note increases in lifespan of both N2 and *adr-1*; *adr-2* in the presence of dsRNA for *daf-2*. The 50% survival time in the *adr-1*; *adr-2* mutant animals was 10 days (95% limits 9 and 12 days) compared with 20 days (95% limits 18 and 20 days) for N2 wild-type control worms. RNAi to *daf-2* increases lifespan to 34 days (95% limits 32 and 40 days), compared with 20 days for the wild type (N2 worms fed empty vector (RNAi)). *daf-2* gene inactivation, in the background of the *adr-1* and *adr-2* null mutations also restored lifespan to 18 days (95% limits 16 and 20 days), compared with 10 days for the *adr-1;adr-2* double mutant strain. b) Lifespan using mutant strains for *adr-1;adr-2* (solid red), N2 wildtype (solid blue), *rde-1* (grey hatched), *adr-1*; *adr-2*; *rde-1* (grey solid) demonstrate declines in lifespan using mutant strains and full rescue of lifespan in an RNAi defective (*rde-1*) background. The *adr-1*; *adr-2* mutant was again about half as long lived as wild-type (median survival time 9 days for *adr-1;adr-2* strain (95% limits 9 and 11 days), and median survival time 21 days (95% limits 18 and 21 days), for N2 wild-type worms. The survival distribution of the triple mutant *adr-1;adr-2*; *rde-1* is median lifespan 21 days (95% limits 18 and 21 days), which is significantly different from *adr-1;adr-2*, with a median lifespan of 9 days (95% limits 9 and 11 days). The lifespan of *rde-1* was modestly reduced compared with the wild-type N2, as was reported previously^29^. Inset boxes displays 50% survival (days) for each condition and demonstrates that daf-2 gene inactivation increases lifespan, in both wild type and in *adr-1;adr-2* mutant strains (a) and that RNAi knockout (*rde-1*) restores lifespan.

To go beyond statistical association, we chose to investigate the possible functional role of these genes in regulating lifespan by evaluating their influence on lifespan in the nematode, *C. elegans*, a robust model organism for candidate lifespan gene discovery. The A-to-I RNA editing gene family and their enzymatic editing activity has been well conserved in a broad array of species including humans, mice, flies, zebrafish, xenopus and notably, *C. elegans* [31], [35]. For lifespan analysis, we focused on *C. elegans*, which has two orthologues of *ADARB1* and *ADARB2* with RNA editing activity, *adr-1* and *adr-2* [36] (see phylogenetic tree in Figure S1). Because *C. elegans* has an average lifespan of approximately 20 days, the influence of candidate genes on lifespan can be readily tested. Therefore, to evaluate whether *adr* loss-of-function influences *C. elegans* lifespan, we monitored the lifespan of single and double mutants of *adr-1(gv6)* and *adr-2(gv42)*. Both of these alleles are deletions that remove at least a third of the coding sequence and are presumed null alleles [36] (Figure 6a and Figure S2). Strains carrying mutations in *adr-1* and *adr-2* displayed a shorter lifespan than the wild-type control N2 worms (log-rank test p<10^−8^). Remarkably, aside from the decline in lifespan, there were no other obvious defects, in contrast with gain-of-function studies that noted lethality in Drosophila [37].

The insulin-like growth factor (IGF) pathway is a well known lifespan regulatory axis in worms [38], flies [39], [40], mice [41], and humans [42]. Inactivation of the *C. elegans* insulin like receptor gene *daf-2* by dsRNA increased lifespan of both the wild type N2 worms, confirming previous studies. Notably, knockdown of *daf-2* by dsRNA also increased lifespan in the background of *adr-1* and *adr-2* null mutations, resulting in a lifespan phenocopy similar to the wild type N2 worms (Figure 6a). Similar extensions of lifespan were seen with single *adr-1* or *adr-2* mutants, (Figure S2). These data suggest that IGF axis mediated increases in lifespan due to *daf-2* remain active in the presence of *adr-1 and adr-2* background genotypes, but with less potency than in a N2 wild type background. From these data, we cannot exclude the possibility that knockdown of RNA editing genes in *adr-1* or *adr-2* mutants results in increased RNAi activity. In this scenario, increased RNAi might target genes downstream of *daf-16* (seven *daf-16* gene targets have been identified in comparative analysis of daf-2 versus *daf-2::daf-16* strains [43]), thereby reducing the potency of *daf-2* dependent increases in lifespan.

In a previous study, mutations in both *adr-1* and *adr-2* resulted in increased GFP reporter transgene silencing, suggesting that declines in ADAR function are associated with an increase in RNA interference (RNAi), which would account for the GFP silencing in those experiments. When the argonaute gene *rde-1*, which is essential for RNA induced silencing complex (RISC) formation, was introduced into *adr-1;adr-2* worms containing the transgenes, the increased GFP silencing due to ADAR knockdown was suppressed [44]. This suggests cross-regulation between RNA editors and RNA interference that is further supported by results from Tonkin et al [45], wherein they demonstrated that a mild chemotaxis defect of the *adr-1;adr-2* double mutant could be rescued by an *rde-1* mutant [45]. Therefore, to evaluate the potential for cross-regulation between RNA editing and RNA interference in the context of lifespan, we evaluated *adr-1*; *adr-2* mediated declines in lifespan in the presence of the RNAi defective strain, *rde-1(ne-219)*. Remarkably, and consistent with previous results, the loss of *rde-1* completely restored lifespan declines associated with *adr-1*; *adr-2* loss-of-function (Figure 6b). We interpret these data as expanding the interaction between these two RNA regulatory pathways to include lifespan determination.

Our experiments with *C. elegans* raise the question as to the precise role for A-to-I RNA editing gene activity in human aging, which remains unknown. However, the demonstration that this family of genes is implicated in the regulation of aging in other organisms warrants validation in other species, particularly humans, and provides a novel regulatory axis for future studies on regulatory pathways that influence the aging process. We speculate that as of yet unidentified ADAR variants delay age associated declines in ADAR activity. We note that a reduction in ADAR enzymatic activity is associated with Dementia, ALS and Alzheimers disease in normal aging individuals [46]. Consistent with this interpretation, analysis of published transcriptional profiles of aging in C. elegans indicate that *adr-1 and adr-2* expression peak in early adulthood and decline with age rather precipitously. The declines observed in that study are compatible with a protective role for ADAR alleles in aging.

Prior to this study, RNA editing had not been directly implicated in the regulation of aging in humans or *C. elegans*. Although the impact upon lifespan that we observed in *C. elegans* appears to be independent of insulin signaling (Figure 6a), the interaction between RNA editing and RNA interference is likely to be complex since decreased insulin signaling in *C. elegans* can also affect RNA interference [47] and may suggest threshold effects associated with declining levels of insulin signaling. Future studies will be needed to identify and characterize targets of RNA editing and their potential role(s) in modulating RNA interference activity, in the context of aging and age-related diseases.

Our analysis provides strong evidence for association of *ADARB1* and *ADARB2* with extreme old age. Our findings of strongest association in the NECS sample are consistent with that sample being both the largest and oldest of the four studies. The lack of reproducibility for some SNPs may have been due to differences in overall genetic background (ethnicity), size and younger ages of the oldest old samples. Nonetheless, associations were noted across four different study populations suggesting that the associations between *ADARB1* and *ADARB2* and EL are robust to different genetic backgrounds and environmental exposures. *ADARB2* is a very large gene spanning more than 500Kb in chromosome 10, but our analysis narrows the association to a region of approximately 100Kb that could be followed-up by fine mapping or sequencing for discovering functional variants and to provide a better understanding of the function of these genes in human aging.

## Materials and Methods

### Ethic Statement

Subjects included in the NECS, SICS, AJCS and JCS provided written informed consent, and all research involving human subjects was approved by the Institutional Review Boards of Boston University, Boston, USA (NECS), the “Istituto di Ricovero e Cura a Carattere Scientifico “Multimedica, Milano, Italy (SICS), Albert Eistein College of Medicine, Bronx, USA (AJCS), Keio University, Tokyo, Japan (JCS). All data were analyzed anonymously. Control data from the Illumina iControlDB database were anonymized.

### SNP Genotyping

For the NECS and SICS samples, 1 ug of genomic DNA was analyzed on the Illumina 370 CNV chip (Illumina, San Diego, CA) and only samples with at least a 93% call rate were used for the analysis. For the AJCS and JCS, genotyping was originally performed with the Affymetrix 6.0 chip and 5.0 chips, respectively with required call rates of 99% or greater. Affymetrix Birdseed algorithm and Illumina Beadstudio were used for genotype calling. Non overlapping SNPs that were not approximated by SNPs with substantial LD (D′>0.8) were genotyped with Sequenom (AJCS) and BigDye Terminator cycle sequencing kit and an ABI Prism 3730xl DNA analyzer (Applied Biosystems, CA, USA). The sequence data were analyzed with ABI PRISM SeqScape Software version 2.6 (Applied Biosystems).

### Creation of a Genetically Matched Control Set

A referent cohort sample for the NECS subjects was constructed utilizing genotype data from the Illumina iControlDB database and principal components analysis was used to match cases and controls by genetic background. To reduce chances of stratification, we identified 2,077 Caucasian referent subjects from the Illumina iControlDB, all genotype with Illumina arrays, with known age at enrollment between 30 and 75 years, and we used the principal component analysis implemented in the program EIGENSTRAT [48] to examine the structure of this referent group compared to the NECS and SICS subjects. The analysis showed that both the NECS and the Illumina controls are comprised of three major clusters that correspond to northwest, northeast and southwest Europeans, but in the Illumina controls sample there were also subjects with different levels of admixture between the three clusters Figure S3. We therefore randomly sampled 1,538 subjects from the three major clusters to create a control set that matched the genetic background of the NECS extreme old sample set as suggested in [49]. We use the same procedure to identify 81 female and 16 male subjects to be added to the set of SICS controls. The random-selection procedure was repeated twice and lead to the same results.

### Genetic Association Analysis (Pooled DNA Samples)

The statistical analysis of pooling based genome-wide genotype data is described in [27]. Briefly, the method uses Bayesian association tests to score the evidence for allelic associations between centenarians and controls. Prior distributions represent the prior knowledge about the expected number of genes that may be implicated with the trait and therefore correct for multiple comparisons. The analysis also uses linkage disequilibrium (LD) based filters to retain associations that are supported by clusters of SNPs in LD.

### Genetic Association Analysis (Individual DNA Samples)

The genotype data of the 31 SNPs in the genes *ADARB1* and 114 SNPs in *ADARB2* were individually analyzed using Bayesian logistic regression [32] to fit dominant and recessive models of inheritance adjusted by gender. The marginal posterior distributions of the ORs were estimated using the implementation of Gibbs sampling in WinBugs 1.4 [50], and the 2.5^th^ and 97.5^th^ percentile were used to estimate 95% credible intervals (CI) for the ORs. The 50^th^ percentile was used to estimate the OR, and the frequency of OR>1 was used to estimate the posterior probability p(OR>1). The Gibbs sampler was run for at least 10,000 iterations and the last 10,000 simulated values were used to estimate these parameters. We used as prior distributions on the regression coefficients of the logit function normal distributions with mean 0 and standard deviation 3.2 that determine a normal prior distribution of the log(OR) with mean 0 (no association) and a variance that ranges between 10 with no genetic effect to 40 with a gene×gender effect. This set of prior distributions was determined to make the analysis robust to rare alleles (frequency<0.10) and we searched for the largest variance that allowed successful execution of the Gibbs sampler. These prior distributions bias the analysis toward the null hypothesis and reduce false positive associations.

The Bayes significance was defined as 1- p(OR>1) when the posterior estimate of the OR was >1, and 1-p(OR<1) when the posterior estimate of the OR was <1, and an association was deemed significant in the NECS, SICS, or the data aggregated from the two studies, if the Bayes significance was smaller than 0.05. This measure of significance is the posterior probability of the null hypothesis OR≤1 (or OR≥1) so that small values denotes strong evidence against the null hypothesis [32]. This analysis identified 18 significant SNPs (Table 3), that is more than twice the number expected by chance in 145 independent tests and two SNPs remained significant even after correcting the threshold for the number of tests. Furthermore, the probability that 18 SNPs could be simultaneously found significantly associated under the null hypothesis of no association is 0.0002, using the binomial distribution with n = 145, x = 18 and p = 0.05. An association that was significant in the aggregated NECS and SICS data was deemed replicated in either the AJCS or JCS studies if the same SNP was significant (Bayes significance <0.05) with the same genetic model and consistent effects; or the same SNP did not reach statistical significance (Bayes significance ≥0.05), but the ORs in the different studies were in the same direction and when the data from the studies were aggregated, the association was significant. The rationale for the second condition is that both the AJCS and JC have smaller sample sizes, and therefore have less power compared to the NECS. However, consistent effects and increased significance when the aggregated data are analyzed show that the lack of association in the replication study is due to lack of power if effects are similar across different studies. This strategy has been used to increase the power of genetic association studies, see for example [51]. The results are in Tables 4, 5, 6 and 7.

We conducted a similar analysis stratified by gender but the limited sample sizes did not produce strongly significant results.

### Linkage Disequilibrium (LD) Heatmaps

We used HaploView 4.1 to create the LD heatmaps and LD displays were generated using the D′ color scheme where white represents D′ = 0, red represents D′ = 1, and different shades of red represent 0<D′<1 (Figures 1 and 4).

### Lifespan Measurements in C. elegans

To synchronize worms for lifespan, eggs were isolated (*N2*, *adr-1*, *adr-2*, *adr-1;adr-2*, *rde-1*, *rde-4*, *adr-1;adr-2;rde-1*) and synchronized by hatching overnight in the absence of food at 20C. Synchronized L1 larvae were counted and plated (10 worms/plate, n = 60) on Escherichia coli bacterial lawns (OP50) on NGM media and allowed to develop to L4-stage larvae at 20C. 5-fluorodeoxyuridine (FudR) solution was added to a final concentration of 0.1mg/ml to prevent reproduction. Worms were kept at 20C and lifespan monitored by counting on alternate days. Lifespan was defined as the first day of adulthood (adult lifespan = 0) to death. Aside from reduced lifespan, the worms appeared normal in size and general behavior, consistent with previous reports on *adr* mutant strains [36]. We did observe altered chemotaxis during routine passage of the worms to maintain stocks, as previously noted [36]. We also noted reduced progeny viability (data not shown). However, these are unlikely to have influenced our lifespan measurements, since the adult worms were made sterile using FudR and were not transferred during the course of the lifespan assay.

### RNAi and Lifespan Measurement

Eggs were isolated from gravid worms and synchronized by hatching overnight in the absence of food. The synchronized L1 larvae were then placed on OP50-containing agar plates and allowed to develop to L4-stage larvae at 20C. The L4-stage larvae were washed thoroughly, and placed either on *Escherichia coli* HT115 with empty RNAi vector or *Escherichia coli* HT115 expressing double-stranded RNA (dsRNA) for *daf-2*. Briefly, dsRNA -expressing bacteria were grown overnight in LB with 50 ug/ml ampicillin and then seeded onto RNAi NGM plates containing 5 mM isopropylthiogalactoside (IPTG). The RNAi bacteria were induced overnight at room temperature for dsRNA expression. About 30 synchronized L4-stage animals were added to each well and allowed to develop to adults, followed by the addition of FudR. Worms were kept at 20C, and their lifespan was monitored. Worms feeding on bacteria carrying the empty vector were used as a negative control. Log-rank test in the R package survival was used for the statistical analysis.

## Supporting Information

Figure S1 Phylogenetic clustering and alignment of ADARs form multiple species. Phylogenetic clustering and alignment of ADARs form multiple species, including Human, chimp, bull, cat, rat, chicken, wolf and nematode (C. elegans). Sequences were aligned by using the neighbor-joining algorithm with Clustal X, gap-stripped with corrections for multiple substitutions, and bootstrap analyzed with 1,000 bootstrap resamplings. Phylograms were generated with NJ-plot. The tree is unrooted and was generated using a Neighbor Joining algorithm implemented in Clustal X. Note bootstrap values defining the three main ADAR branches.
(0.07 MB TIF)

Figure S2 C. elegans lifespan results using individual gene mutant strains. C. elegans lifespan results using individual gene mutant strains, as indicated. Eggs were isolated from gravid worms and synchronized by hatching overnight in the absence of food. The synchronized L1 larvae were then placed on OP50-containing agar plates and allowed to develop to L4-stage larvae at 20C. The L4-stage larvae were washed thoroughly, and placed on Escherichia coli expressing double-stranded RNA (dsRNA) for daf-2. Briefly, dsRNA -expressing bacteria were grown overnight in LB with 50 ug/ml ampicillin and then seeded onto RNAi NGM plates containing 5 mM isopropylthiogalactoside (IPTG). The RNAi daf-2 bacteria were induced overnight at room temperature for daf-2 dsRNA mediated knockdown expression. About 30 synchronized L4-stage animals were added to each well and allowed to develop to adults, followed by the addition of FudR. Worms were kept at 20C, and their lifespan was monitored. Worms feeding on bacteria carrying the empty vector were used as a negative control. Animals were scored every 1 to 2 days subsequently and scored as dead when they no longer responded to gentle prodding.
(0.05 MB TIF)

Figure S3 Population structure of NECS centenarians and controls, SICS centenarians and controls, and Illumina controls. Population structure of NECS centenarians (blue) and controls (red), SICS centenarians (green) and controls (orange), and Illumina controls (grey). Each scatter plot shows the first two principal components that were estimated using genotype data for more than 300K SNPs in NECS, SICS and Illumina subjects using the program Eigenstrat. From top to bottom, left to right: (Blue) scatter plot of the first two principal components in centenarians of the NECS. The two principal components (PC1 displayed in the x-axis and PC2 in the y-axis) identify 3 major clusters that based on the ancestry of the NECS centenarians can be labeled as NW Europeans (PC1<0.005 and −0.0125<PC2<0.0125), Ashkenazi Jews (PC1>0.005 and PC2<−0.0125) and SW Europeans/Italians (PC1>0.005 and PC2>0.0125). The thresholds on the principal components were identified by splitting the components using mixture models. (Green) scatter plot of the first two principal components in centenarians of the SICS. In agreement with the analysis of NECS subjects, the centenarians of the SICS have a SW European genetic background. (Red) scatter plot of the first two principal components in controls of the NECS that display approximately the same population substructure of centenarians; (Grey) scatter plot of the first two principal components in the Illumina controls. The plots show that the controls have a population substructure similar to the NECS cases and controls but also a larger level of admixture between the three European subgroups. (Orange) scatter plot of the first two principal components in controls of the SICS that exhibit the same SW European genetic background of SICS centenarians. Note that the plot of PC1 and PC2 for NECS controls (red) is repeated twice to facilitate the two comparisons within NECS subjects and between NECS and Illumina controls.
(0.12 MB TIF)
