# Correction: RNA Editing Genes Associated with Extreme Old Age in Humans and with Lifespan in *C. elegans*

**DOI:** 10.1371/annotation/387f8074-5f80-4bdd-bb0b-b36d49a16ac0

**Published:** 2009-12-15

**Authors:** Paola Sebastiani, Monty Montano, Annibale Puca, Nadia Solovieff, Toshio Kojima, Meng C. Wang, Efthymia Melista, Micah Meltzer, Sylvia E. J. Fischer, Stacy Andersen, Stephen H. Hartley, Amanda Sedgewick, Yasumichi Arai, Aviv Bergman, Nir Barzilai, Dellara F. Terry, Alberto Riva, Chiara Viviani Anselmi, Alberto Malovini, Aya Kitamoto, Motoji Sawabe, Tomio Arai, Yasuyuki Gondo, Martin H. Steinberg, Nobuyoshi Hirose, Gil Atzmon, Gary Ruvkun, Clinton T. Baldwin, Thomas T. Perls

There was an error in affiliation assignment for author Annibale Puca. This author should also be affiliated with: Istituto di Tecnologie Biomediche - Consiglio Nazionale delle Ricerche, Segrate, Italy. 

