# Supplementary figures and images for: RNA Editing Genes Associated with Extreme Old Age in Humans and with Lifespan in C. elegans
Source: PLoS One. 2009 Dec 14;4(12):e8210. doi: 10.1371/journal.pone.0008210 (PMC2788130; doi:10.1371/journal.pone.0008210)

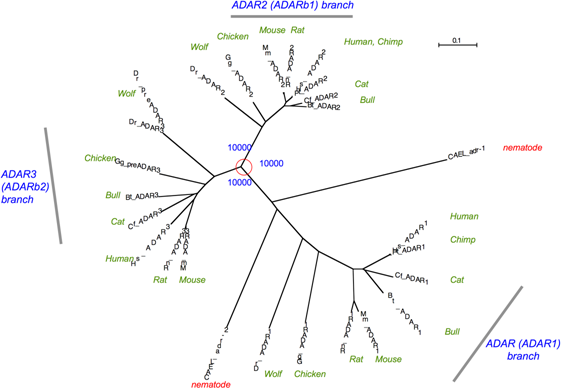

Supplement: Figure S1 — Phylogenetic clustering and alignment of ADARs form multiple species. Phylogenetic clustering and alignment of ADARs form multiple species, including Human, chimp, bull, cat, rat, chicken, wolf and nematode (C. elegans). Sequences were aligned by using the neighbor-joining algorithm with Clustal X, gap-stripped with corrections for multiple substitutions, and bootstrap analyzed with 1,000 bootstrap resamplings. Phylograms were generated with NJ-plot. The tree is unrooted and was generated using a Neighbor Joining algorithm implemented in Clustal X. Note bootstrap values defining the three main ADAR branches. (0.07 MB TIF) [file pone.0008210.s001.tif]

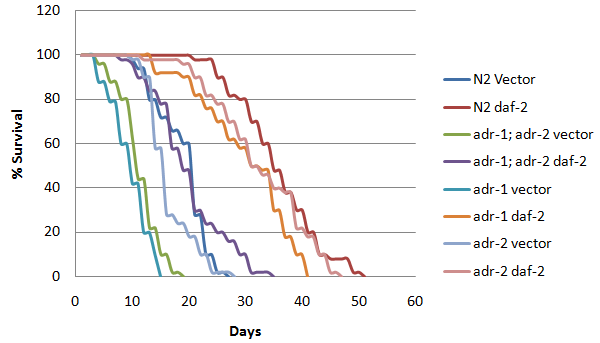

Supplement: Figure S2 — C. elegans lifespan results using individual gene mutant strains. C. elegans lifespan results using individual gene mutant strains, as indicated. Eggs were isolated from gravid worms and synchronized by hatching overnight in the absence of food. The synchronized L1 larvae were then placed on OP50-containing agar plates and allowed to develop to L4-stage larvae at 20C. The L4-stage larvae were washed thoroughly, and placed on Escherichia coli expressing double-stranded RNA (dsRNA) for daf-2. Briefly, dsRNA -expressing bacteria were grown overnight in LB with 50 ug/ml ampicillin and then seeded onto RNAi NGM plates containing 5 mM isopropylthiogalactoside (IPTG). The RNAi daf-2 bacteria were induced overnight at room temperature for daf-2 dsRNA mediated knockdown expression. About 30 synchronized L4-stage animals were added to each well and allowed to develop to adults, followed by the addition of FudR. Worms were kept at 20C, and their lifespan was monitored. Worms feeding on bacteria carrying the empty vector were used as a negative control. Animals were scored every 1 to 2 days subsequently and scored as dead when they no longer responded to gentle prodding. (0.05 MB TIF) [file pone.0008210.s002.tif]

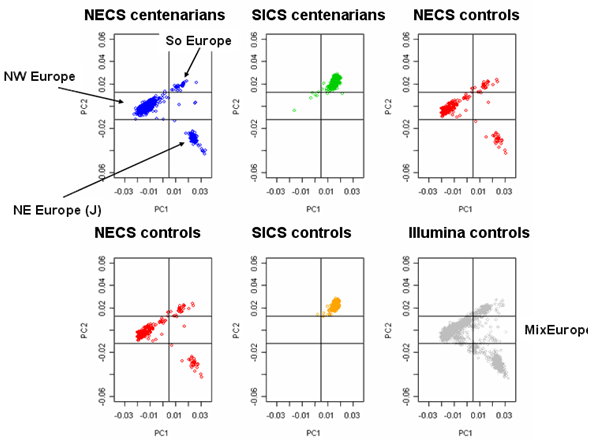

Supplement: Figure S3 — Population structure of NECS centenarians and controls, SICS centenarians and controls, and Illumina controls. Population structure of NECS centenarians (blue) and controls (red), SICS centenarians (green) and controls (orange), and Illumina controls (grey). Each scatter plot shows the first two principal components that were estimated using genotype data for more than 300K SNPs in NECS, SICS and Illumina subjects using the program Eigenstrat. From top to bottom, left to right: (Blue) scatter plot of the first two principal components in centenarians of the NECS. The two principal components (PC1 displayed in the x-axis and PC2 in the y-axis) identify 3 major clusters that based on the ancestry of the NECS centenarians can be labeled as NW Europeans (PC1<0.005 and −0.0125<PC2<0.0125), Ashkenazi Jews (PC1>0.005 and PC2<−0.0125) and SW Europeans/Italians (PC1>0.005 and PC2>0.0125). The thresholds on the principal components were identified by splitting the components using mixture models. (Green) scatter plot of the first two principal components in centenarians of the SICS. In agreement with the analysis of NECS subjects, the centenarians of the SICS have a SW European genetic background. (Red) scatter plot of the first two principal components in controls of the NECS that display approximately the same population substructure of centenarians; (Grey) scatter plot of the first two principal components in the Illumina controls. The plots show that the controls have a population substructure similar to the NECS cases and controls but also a larger level of admixture between the three European subgroups. (Orange) scatter plot of the first two principal components in controls of the SICS that exhibit the same SW European genetic background of SICS centenarians. Note that the plot of PC1 and PC2 for NECS controls (red) is repeated twice to facilitate the two comparisons within NECS subjects and between NECS and Illumina controls. (0.12 MB TIF) [file pone.0008210.s003.tif]
